# Prospecting the Resilience of Several Spanish Ancient Varieties of Red Grape under Climate Change Scenarios

**DOI:** 10.3390/plants11212929

**Published:** 2022-10-31

**Authors:** María Carmen Antolín, Eduardo Salinas, Ana Fernández, Yolanda Gogorcena, Inmaculada Pascual, Juan José Irigoyen, Nieves Goicoechea

**Affiliations:** 1 Plant Stress Physiology Group (Associated Unit to CSIC, EEAD, Zaragoza), Universidad de Navarra-BIOMA, 31008 Pamplona, Spain; 2 Genomics of Fruit Trees and Grapevine Group, Estación Experimental de Aula Dei (EEAD), Consejo Superior de Investigaciones Científicas (CSIC), 50059 Zaragoza, Spain

**Keywords:** phenolic maturity, high temperature, old grapevine varieties, soluble solids, technological maturity, water deficit

## Abstract

Background: Climate change results in warmer air temperatures and an uncertain amount and distribution of annual precipitations, which will directly impact rainfed crops, such as the grapevine. Traditionally, ancient autochthones grapevine varieties have been substituted by modern ones with higher productivity. However, this homogenization of genotypes reduces the genetic diversity of vineyards which could make their ability to adapt to challenges imposed by future climate conditions difficult. Therefore, this work aimed to assess the response of four ancient grapevine varieties to high temperatures under different water availabilities, focusing on plant water relations, grape technological and phenolic maturity, and the antioxidant capacity of the must. Methods: The study was conducted on fruit-bearing cuttings grown in pots in temperature-gradient greenhouses. A two-factorial design was established where two temperature regimes, ambient and elevated (ambient + 4 °C), were combined with two water regimes, full irrigation and post-veraison deficit irrigation, during fruit ripening. Results: There were significant differences among the ancient varieties regarding plant water relations and fruit quality. Conclusion: This research underlines the importance of evaluating the behavior of ancient grapevine varieties that could offer good options for the adaptation of viticulture to future climate conditions.

## 1. Introduction

According to the Intergovernmental Panel on Climate Change [1], climate change is caused mainly by the increase in the concentration of atmospheric carbon dioxide (CO_2_) due to anthropogenic emissions. Under this situation, associated variables determining the weather are also modified, resulting in both warmer air temperatures and an uncertain amount and distribution of annual precipitation. The IPPC data recorded in 2022 indicate that, in the Mediterranean area, air temperature and heat waves will increase throughout the 21st century above the global average, precipitation will decrease between 4 and 22%, episodes of torrential rains will increase in the northern part and droughts will be more prevalent in most areas. As a result, the increased risk of unexpected drought periods will impact directly most cultivated species, mainly in rainfed crops, such as the grapevine [2,3]. Air temperature is one of the most relevant factors in controlling grapevine development. Alterations in the grapevine growth and physiology, associated with increasing temperatures, have been documented [4], with the acceleration of phenology as one of the main outcomes, which leads to the advancement of the ripening of berries to warmer months [5,6]. Moreover, high temperatures alter berry metabolism and composition, which results in red wines with enhanced alcohol levels [7], low acidity, and modified phenolic compositions [8,9,10]; all these changes produce noteworthy modifications of organoleptic properties that distinguish each variety [11,12]. 

In southern Europe, the grapevine has been traditionally grown under rainfed conditions, thus leading to plants with low yield and low vigor but with high fruit quality. However, the growing episodes of water scarcity associated with climate change are modifying this procedure and the area of irrigated vineyards has exponentially increased in the last two decades to mitigate the negative impact of increasingly severe and unpredicted droughts on grapevine quality [2,3]. For instance, in Spain, the irrigated surface of vineyards has passed from 2% in 1950 up to 32% in 2021 [13]. Irrigation of grapevines allows for an increase in yield and mitigates drought, so irrigation is an important tool to reduce the temperature of the canopy, mainly under heat waves, which is becoming an acute constraint for vineyards in the Mediterranean region [4]. However, it is well known that grape berry quality, particularly for red wines, benefits from mild to moderate water deficit conditions [14]. Water deficit reduces canopy vigor, improves fruit exposure to light, and reduces berry growth, which prevents the dilution effects of berry compounds [15]. It has been shown that water deficit modifies the metabolism of the grape berry, promoting the synthesis of volatile and phenolic compounds [16,17,18,19,20], which results in red wines with higher concentrations of total phenols and anthocyanins and more appreciated sensory properties [12,21,22]. There is much literature on the response of vine cultivation and the characteristics of the grape to the increase in temperature and water deficit, separately. However, at present, there are few studies about the combined effect of elevated temperature and deficit irrigation on grape quality [23,24]. 

Given the climate change predictions of increasing air temperatures and more frequent drought episodes, the selection of genotypes with better adaptability to environmental changes will be of great interest for perennial crops, such as grapevine [25]. Traditionally, the autochthone grapevine varieties have been substituted by international cultivars, reducing the genetic diversity of vineyards [26] and leading to the disappearance of numerous minor and ancient genotypes [27]. However, this situation has begun to change, and producers, wineries, and consumers are searching for new products developed with local resources. In Spain, there is a growing interest in identifying and recovering old and forgotten vine varieties to know the existing regional genetic diversity [27,28,29,30]. These minor varieties with limited regional distribution make it possible to obtain wines with typicities that identify specific geographic regions [31]. In addition, this trend attempts to counteract the low diversity in the vineyards and could be a successful alternative to increase the variety of products (grape or wine) in the markets. In old vine varieties, the agronomic and oenological evaluation will constitute an important resource to provide viticulturists with a deeper knowledge of their distinctive characteristics, which will allow the wine market to be enriched and diversified with different products [32]. Alternatively, these varieties could be used in wine blending (coupage) to counteract the negative effects of climate change on the oenological and quality properties of recognized regional wine varieties. Nevertheless, the adaptive responses of such ancient vine varieties need to be further studied, to characterize climate-resilient cultivars. Recent studies point out that those ancient varieties might have a strong potential for adapting to the challenges imposed by climate change, making it an interesting option for cultivation in future conditions [33,34]. Such studies suggest that is needed to conserve the existing biodiversity of autochthonous grapevine genotypes, as some varieties could perform well in future climates, thereby opening new adaptation opportunities [35,36]. Considering all these precedents, the aim of the current research was to assess the response of four ancient grapevine varieties to high temperatures under different water availabilities, focusing on plant water relations, berry technological maturity parameters (i.e., sugars and titratable acidity), phenolic maturity (i.e., quantity and quality of phenolic compounds in skin and seeds), and grape antioxidant capacity. Potted vines were used to ensure that all the varieties were subjected to the same temperatures and controlled water-deficit levels.

## 2. Results

### 2.1. Plant Physiological Characteristics

**Figure 1:**
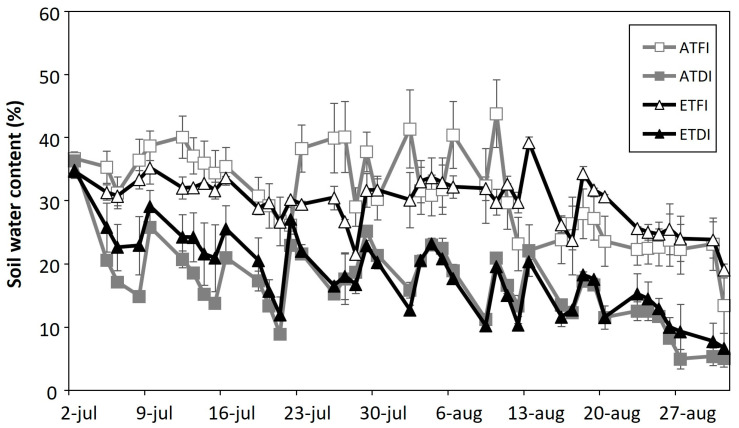
Soil water content measured in the pots every day from veraison to berry maturity. Each point is the mean ± S.E of 3 measurements done in the substrate of plants subjected to either at ambient (AT) or elevated temperature (ET) and subjected to different irrigation regimes (FI, full irrigation; DI, deficit irrigation).

The volumetric soil water content was monitored from veraison to berry maturity in the pots of the full irrigation (FI) and deficit irrigation (DI) treatments (Figure 1). Data of the FI were quite stable, being around 35%, whereas the values of the DI pots oscillated because plants did not receive any water until their soil water content was decreased to 15–20%, the time when the pots were irrigated. 

**Figure 2:**
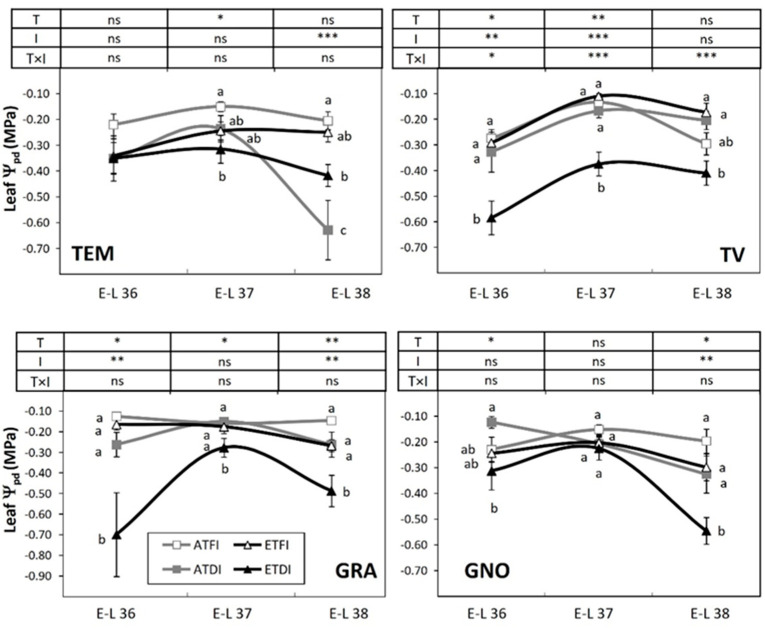
Pre-dawn leaf water potential (Ψ_pd_) recorded at three stages of berry ripening in fruit-bearing cuttings of ancient wine grape genotypes grown either at ambient (AT) or elevated temperature (ET) and subjected to different irrigation regimes (FI, full irrigation; DI, deficit irrigation). Values are means ± S.E. (*n* = 5). Means within each stage followed by the same letter do not differ significantly (*p* > 0.05) according to Duncan’s test. Two-way analyses of variance (ANOVA) assess the main effect of the temperature (T), irrigation (I), and their interaction (T × I). Significance of the ANOVA: * *p* < 0.05; ** *p* ≤ 0.01; *** *p* ≤ 0.001; ns, not significant (*p* ≥ 0.05). The variety labels can be found in Table 4.

Four genotypes with oenological potential were chosen for the study (Table 1). Irrigation treatments caused significant differences in vine water status across varieties, as indicated by the decrease in predawn leaf Ψ_pd_ measured in plants subjected to DI from veraison to maturity compared with full-irrigated plants (Figure 2). Under DI, in Tempranillo (TEM) and Grand Noir (GNO), the lowest values of leaf Ψ_pd_ were recorded at the maturity (E-L 38 stage) for ambient (ATDI) and elevated (ETDI) temperature, respectively. The elevated temperature also modified plant water status this factor being especially relevant in Graciano (GRA) and Tinto Velasco (TV), in which Ψ_pd_ was always significantly lower in plants subjected to ETDI compared with ATDI.

Under our experimental conditions, the varieties exhibited remarkable diversity in the length of the ripening cycle and plant growth, as well as bunch and berry traits (Table 2). TEM and GNO were characterized by an earlier onset of veraison than TV and GRA, the latter being the one with the shortest veraison-to-maturity period. Regarding plant growth, TV was the variety with the greatest estimated vegetative development based on leaf area and leaf size. The varieties also differed in some traits related to bunch and berries. In fact, TEM was characterized by a larger bunch, with greater berry mass and seed number per berry than the rest of the varieties. By contrast, TV showed a compact bunch, as well as small berries with low mass (Table 2). 

Regarding the behavior of the grapevine varieties under the climate change scenarios assayed, phenology, plant growth, and most of the fruit characteristics were not affected by increased temperature and/or DI in TEM (Table 3). Only elevated temperature contributed to the increase in the relative skin mass under deficit irrigation (ETDI). In the case of TV, the combination of elevated temperature and DI influenced the maturation time, which was shortened under ETDI. Furthermore, leaf area and bunch mass were significantly reduced at warm temperatures, while the DI treatment resulted in an increase in bunch compactness. The combination of high temperature and DI also affected the GRA variety, so plants subjected to ETDI showed reduced leaf area and a small bunch with a low number and mass of berries. Finally, in GNO, plant growth was reduced in plants subjected to DI, whereas, under elevated temperature, this variety showed a lower number of berries per bunch in ETFI and berries with lower relative skin mass in ETDI. 

### 2.2. Berry Composition

As expected, the variety was the main factor in modifying berry properties and the antioxidant capacity of must, the teinturier GNO being the cultivar with the highest concentration of total anthocyanins, TPI, and antioxidant capacity (Table 4). Altogether, the temperature was the main factor contributing to the increase in must pH and tonality, and to the reduction in total anthocyanins and titratable acidity, while the irrigation increased TSS and must pH. Significant interactions between both factors were detected for titratable acidity (V × I, *p* ≤ 0.01), tonality (V × I, *p* < 0.05), TPI (V × I, *p* < 0.05), and total anthocyanins (V × I, *p* ≤ 0.01). In addition, the effects of high temperature had different intensities depending on the variety, as indicated by the significant interactions found for colour density (V × T, *p* ≤ 0.01), TPI (V × T, *p* ≤ 0.01), total anthocyanins (V × T, *p* ≤ 0.001), and antioxidant capacity (V × T, *p* < 0.05). Similarly, varieties also responded in a different way to DI as highlighted by the significant interaction (V × I) for titratable acidity (*p* ≤ 0.01), tonality (*p* < 0.05), TPI (*p* < 0.05), and total anthocyanins (*p* ≤ 0.01). 

**Figure 3:**
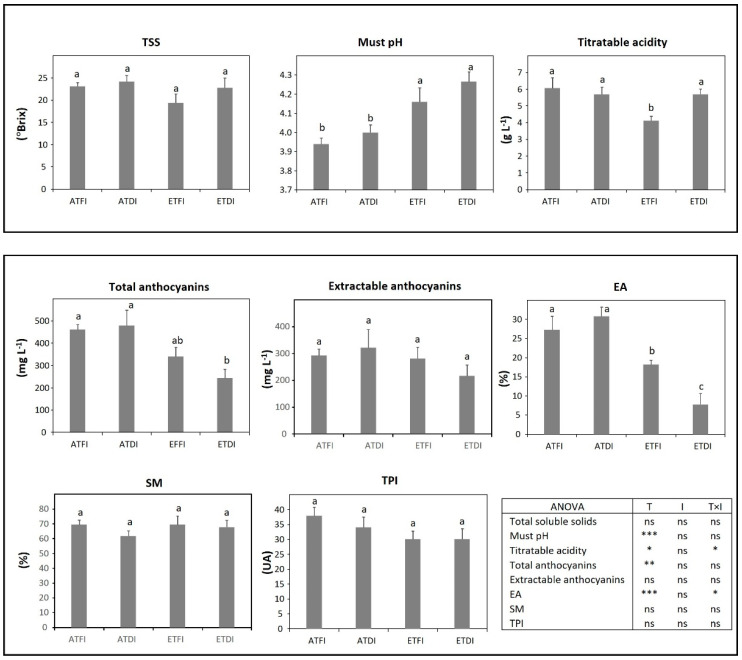
Grape technological and phenolic maturity assessed at harvest in fruit-bearing cuttings of Tempranillo (TEM) grown either at ambient (AT) or elevated temperature (ET) and subjected to different irrigation regimes (FI, full irrigation; DI, deficit irrigation). Values are means ± S.E. (*n* = 5). Bars topped by the same letter do not differ significantly (*p* > 0.05) according to Duncan’s test. Significance of the analysis of variance (ANOVA): * *p* < 0.05; ** *p* ≤ 0.01; *** *p* ≤ 0.001; ns, not significant (*p* ≥ 0.05). TSS, total soluble solids; TPI, total polyphenol index; EA, cellular extractability of anthocyanins; SM, seed maturity; AU, absorbance units.

Regarding the responses of each variety, in TEM, the parameters related to technological maturity were more affected by temperature than by the irrigation level (Figure 3). High temperatures produced an increase in must pH and a decrease in titratable acidity; although, a significant interaction between temperature and irrigation was found (T × I, *p* < 0.05) for titratable acidity. DI, as a single factor, hardly affected the phenolic maturity of the TEM. The temperature was the main factor that contributed to the decrease in total anthocyanins and cellular extractability of anthocyanins (EA), although such effects were more pronounced under water deficit (ETDI) (T × I, *p* < 0.05).

**Figure 4:**
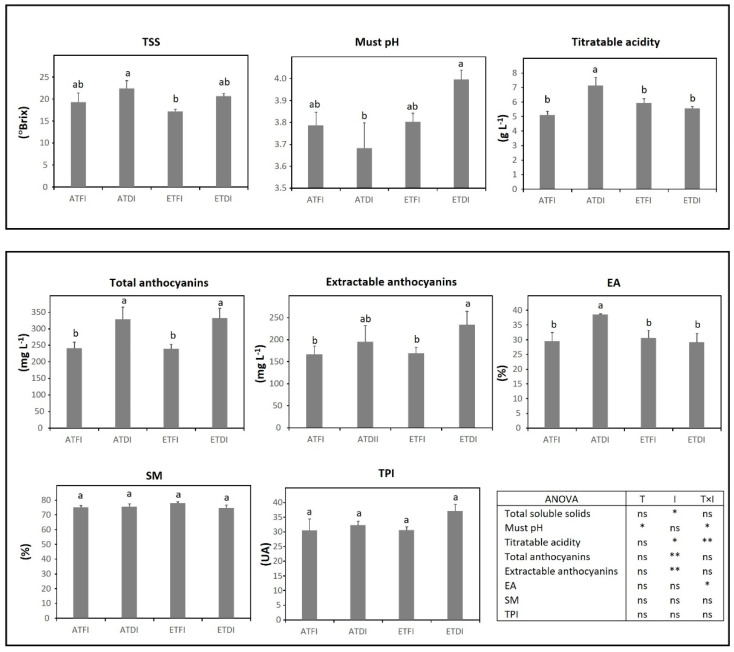
Grape technological and phenolic maturity assessed at harvest in fruit-bearing cuttings of Tinto Velasco (TV) grown either at ambient (AT) or elevated temperature (ET) and subjected to different irrigation regimes (FI, full irrigation; DI, deficit irrigation). Values are means ± S.E. (*n* = 5). Bars topped by the same letter do not differ significantly (*p* > 0.05) according to Duncan’s test. Significance of the analysis of variance (ANOVA): * *p* < 0.05; ** *p* ≤ 0.01; ns, not significant (*p* ≥ 0.05). TSS, total soluble solids; TPI, total polyphenol index; EA, cellular extractability of anthocyanins; SM, seed maturity; AU, absorbance units.

In TV, analyses of technological maturity showed that temperature was the main factor increasing must pH, whereas DI contributed to increased TSS and titratable acidity (Figure 4). The two-way ANOVA analysis showed a significant interaction between temperature and irrigation level for must pH (T × I, *p* < 0.05) and for titratable acidity (T × I, *p* ≤ 0.01). In this variety, phenolic maturity was mainly modulated by irrigation, increasing the accumulation of total anthocyanins under water deficit (ATDI and ETDI treatments) regardless of the temperature regime. The concentration of extractable anthocyanins also improved in those plants grown under ETDI while the highest EA was obtained under ATDI. 

**Figure 5:**
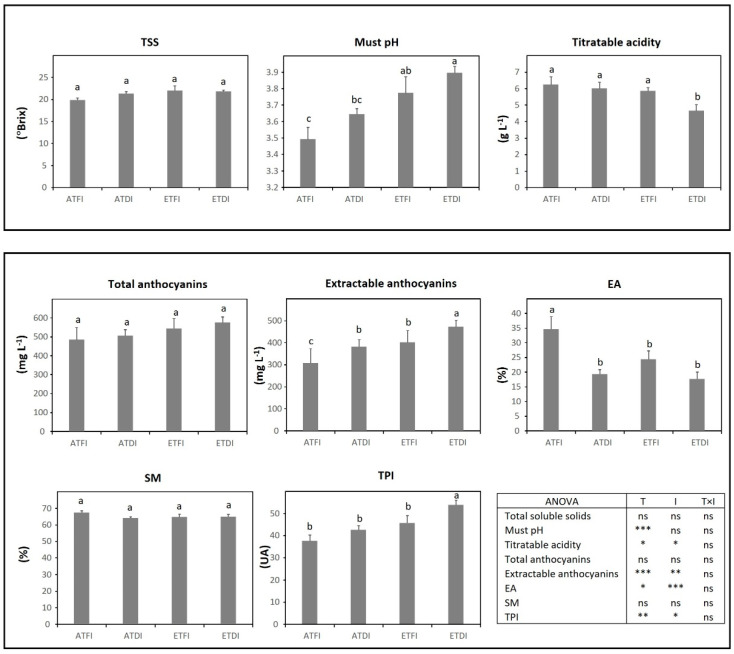
Grape technological and phenolic maturity assessed at harvest in fruit-bearing cuttings of Graciano (GRA) grown either at ambient (AT) or elevated temperature (ET) and subjected to different irrigation regimes (FI, full irrigation; DI, deficit irrigation). Values are means ± S.E. (*n* = 5). Bars topped by the same letter do not differ significantly (p > 0.05) according to Duncan’s test. Significance of the analysis of variance (ANOVA): * *p* < 0.05; ** *p* ≤ 0.01; *** *p* ≤ 0.001; ns, not significant (*p* ≥ 0.05). TSS, total soluble solids; TPI, total polyphenol index; EA, cellular extractability of anthocyanins; SM, seed maturity; AU, absorbance units.

Berry attributes of GRA were clearly modified by both temperature and irrigation, acting alone or in combination (Figure 5). Regarding technological maturity, the temperature was the main factor contributing to the increase in must pH while there were additive effects of both factors for titratable acidity, which gave a significant reduction in this trait under ETDI. Analyses of phenolic maturity indicated that warm temperatures and/or DI enhanced the extractable anthocyanin content by reducing EA. Furthermore, TPI was significantly higher in plants grown under ETDI.

**Figure 6:**
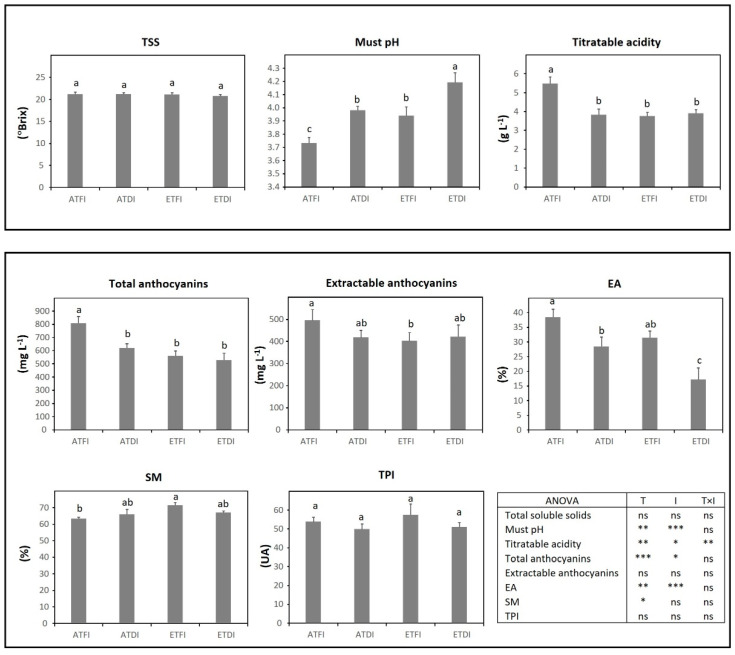
Grape technological and phenolic maturity assessed at harvest in fruit-bearing cuttings of Grand Noir (GNO) grown either at ambient (AT) or elevated temperature (ET) and subjected to different irrigation regimes (FI, full irrigation; DI, deficit irrigation). Values are means ± S.E. (*n* = 5). Bars topped by the same letter do not differ significantly (*p* > 0.05) according to Duncan’s test. Significance of the analysis of variance (ANOVA): * *p* < 0.05; ** *p* ≤ 0.01; *** *p* ≤ 0.001; ns, not significant (*p* ≥ 0.05). TSS, total soluble solids; TPI, total polyphenol index; EA, cellular extractability of anthocyanins; SM, seed maturity; AU, absorbance units.

Concerning GNO, all the treatments with elevated temperature and DI resulted in high must pH, low titratable acidity, and low total anthocyanins (Figure 6), while EA was only reduced in plants subjected to DI. A significant increase in the phenolic maturity of seeds (SM) was found in ETFI conditions.

### 2.3. Antioxidant Capacity

**Figure 7:**
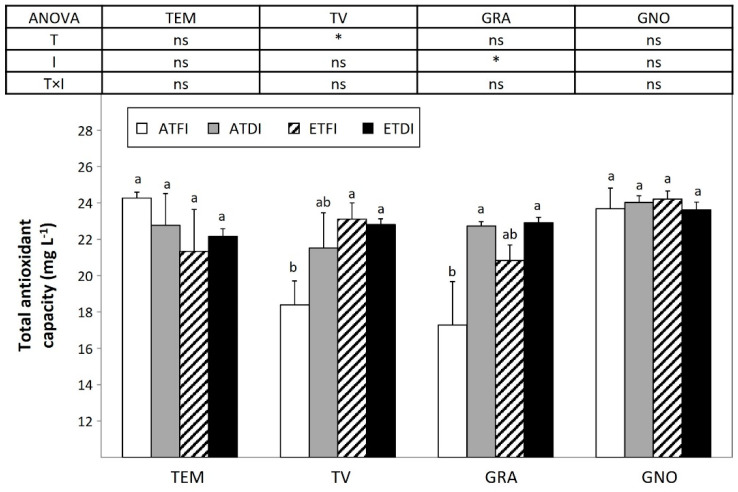
Antioxidant capacity of the must from fruit-bearing cuttings of ancient wine grape genotypes grown either at ambient (AT) or elevated temperature (ET) and subjected to different irrigation regimes (FI, full irrigation; DI, deficit irrigation). Values are means ± S.E. (*n* = 5). Within each variety, bars topped by the same letter do not differ significantly (*p* > 0.05) according to Duncan’s test. Two-way analyses of variance (ANOVA) assess the main effect of the temperature (T), irrigation (I), and their interaction (T × I). Significance of the analysis of variance (ANOVA): * *p* < 0.05; ns, not significant (*p* ≥ 0.05). The variety labels can be found in Table 4.

The total antioxidant capacity was assessed by the mean of the DPPH assay and showed that the temperature rise was the main factor accounting for the enhanced total antioxidant capacity of berry extracts of TV (Figure 7). However, in the case of GRA, the improvement of total antioxidant capacity was restricted to plants subjected to DI regardless of the temperature regime. 

## 3. Discussions

The predicted increase in air temperatures and in the frequency and intensity of droughts are becoming challenges for most of the wine regions of southern Europe [37]. Climate change will induce changes in grapevine physiology and in grape composition that will modify wine typicity [11,12]. However, these modifications can be counteracted through adaptations in the plant material as well as in the viticultural techniques [38]. Concerning plant material, our recent studies supported the idea that ancient grapevine varieties can be useful to cope with the detrimental effects of climate change on grape quality [35,36]. These studies reported that the berry composition significantly varied in some ancient cultivars (including TEM, TV, and GRA) when plants undergo high air temperature combined with elevated atmospheric CO_2_. To find out more about some of these varieties, the present study analyzes their response to deficit irrigation under warm temperatures, in terms of plant water relations, the technological and phenolic maturity of berries, and must antioxidant properties. 

In the last decades, considerable effort has been made to optimize deficit irrigation programs that improve water use and berry quality in vineyards of semiarid areas, with different results depending on the timing, severity, and duration of water deficit [39]. The present study attempts to eliminate such variations by subjecting all varieties to a similar water stress level at post-veraison, for comparison. In our experimental conditions, leaf Ψ_pd_ of plants subjected to DI ranged between −0.3 and−0.8 MPa (Figure 2) and agree with physiological threshold values reported under field conditions in plants subjected to moderate water deficit [39]. The response of plants to drought depends on stomata, which they close to reducing transpiration, avoiding critical Ψ_pd_ and maintaining tissue water content [14]. In our case, except for TEM, the lowest values of leaf Ψ_pd_ were recorded in the ETDI treatment, probably because of the higher vapor pressure deficit, which in turn, could have resulted in increased stomatal conductance and transpiration rates that aided in dissipating heat in leaves by evaporative cooling [40,41]. Concerning the varieties, the lowest leaf Ψ_pd_ measured throughout the course of berry maturation was reached at stage E–L 36 (in TV and GRA) and at stage E–L 38 (in TEM and GNO), suggesting that the former varieties reacted earlier to soil water restriction than the later ones, possibly due to their higher leaf area (Table 2). 

The ancient varieties studied differed in traits such as the length of the reproductive cycle, vegetative growth, bunch structure, and berry characteristics (Table 2). According to our results, TEM stood out for having shorter fruit set to veraison period, higher bunch mass and bigger berries with many seeds, whereas TV was characterized by having higher vegetative development and a compact bunch with small berries. GRA and GNO were characterized by presenting low bunch mass. The increase in air temperature is one of the main factors accelerating grapevine phenology leading to shorter fruit ripening [5,6]. Drought episodes could also produce a higher advance in the veraison and harvest [16,42,43]. However, the impact of climate on grapevine phenology also depends on the genotype [44]. In the present study, neither elevated temperature nor DI modified the length of ripening phases in most varieties (Table 3), suggesting that phenology of ancient varieties could be more resilient to a wide range of climate conditions. Moreover, and accordingly to Biasi et al. [44], the phenological behavior was mainly determined by the genotype as can be seen in the present study, where TV was the only variety, whose ripening time was shortened in the ETDI treatment (Table 3). 

The effects of elevated temperature and water deficit on plant growth and fruit characteristics also differed among varieties (Table 3). In TV and GRA, the bunch and berry traits were more affected by elevated temperature than in GNO and TEM, the latter being the less sensitive variety. It is well known that moderate deficit irrigation decreases plant canopy vigor, reduces berry growth, and increases fruit exposure to light [15]. In the present study, plant growth (as estimated from the leaf area) decreased under DI in GRA and GNO but in the case of TV, plant growth only decreased when DI was combined with high temperature (Table 3). Additionally, DI had little effect on bunch and berry characteristics of most of the varieties, probably because water deficit was applied after veraison when berries have finished their growing phase [45]. GRA was the sole variety in which DI reduced berry mass when combined with high temperature (ETDI) (Table 3), which could lead to high concentrations of phenolic compounds, particularly, anthocyanins [15]. 

Water deficit and high temperature have a great impact on berry composition [10,19]. TSS, the pH and must titratable acidity predict the main technological parameters of wine, such as alcohol content, pH, and total acidity; however, phenolic maturity parameters give information regarding skin and seed phenolic compounds that are key to producing high-quality red wines [46]. Concerning, technological maturity, the accumulation of sugars and the accumulation and degradation of organic acids are modified by high temperature [8,47], which leads to the production of unbalanced red wines with high alcohol levels [7], higher must pH, and low acidity [9]. Our results show that the increase in temperature was the main factor driving the increase in must pH and low titratable acidity. Likewise, DI accounted for increased TSS and must pH (Table 4), which agrees with previous studies [16,17,48]. Regarding titratable acidity, the results of TEM (Figure 3), GRA (Figure 5), and GNO (Figure 6) coincided with the general trend that high temperature and water deficit decreased grape acidity, mainly because of the faster depletion of malic acid [9,14,15]. However, the variety TV only displayed slight changes in pH and titratable acidity (Figure 4), supporting the existent diversity in the responsiveness of the ancient varieties [44]. Regarding TSS, our results indicate that almost all varieties, i.e., TEM (Figure 3), GRA (Figure 5), and GNO (Figure 6) were able to maintain sugar concentrations under a broad range of climatic scenarios. 

Water deficit usually results in red wines with a higher concentration of phenolic compounds (mainly, anthocyanins and flavonols) [21,22], due to altered berry metabolism and the upregulation of several genes of the phenylpropanoid pathway [16,17,19,20]. Nevertheless, this trend could change depending on the variety [18,49] and could be also, conditioned by temperature [23,24]. On the other hand, common high-temperature effects on berry composition include the reduction in anthocyanin content, which leads to diminished total phenolic content of berries [50]. Low anthocyanin concentration under elevated temperature may result from different mechanisms, such as the reduced expression of the genes involved in anthocyanin biosynthesis at veraison [10] or increased enzymatic degradation by peroxidases [47,51]. In our experimental conditions, when the warm temperature was combined with DI (ETDI) total anthocyanin content and TPI were improved in TV (Figure 4) and GRA (Figure 5), respectively, this response being mainly modulated by the irrigation level. By contrast, this treatment, ETDI, reduced the anthocyanin content in TEM (Figure 3) and GNO (Figure 6). In general, except in GRA which showed lower berry size, results were not associated with dilution effects due to changes in berry size or relative skin mass (Table 4). Such results emphasize the existing diversity of responses to climate conditions among ancient cultivars. 

Phenolic maturity includes not only the overall concentration of phenolic compounds but also their structure and capacity to be extracted from grapes during winemaking [52]. Adequate phenolic maturity is acquired when the content of extractable anthocyanins in the skin is high, and the concentration of seed tannins is low [46]. Low values of extractability of anthocyanins (EA) indicate a high percentage of extractable anthocyanins that will pass easily to the wine during fermentation. In our study, EA was decreased by high temperature in TEM (Figure 3), by deficit irrigation in GNO (Figure 6) and by both factors in GRA (Figure 5), which is consistent with a more advanced maturation [38]. In GRA, low EA caused the improvement of the content of extractable anthocyanins regardless of the treatment applied making this variety an interesting option for cultivation in the future climate scenario. By contrast, the phenolic maturity of seeds (SM) was a stable property that only increased in GNO under ETFI, which may involve the increase in the astringency of the resulting wine [53]. 

Phenolic compounds and their antioxidant properties have generated significant interest related to human health benefits [54]. In our study, the antioxidant capacity of the must performed quite differently in function of the variety (Figure 7). In fact, TV showed increased antioxidant capacity at a high temperature (ETFI and ETDI), whereas GRA improved their antioxidant capacity under water deficit (ATDI and ETDI). Consequently, under ETDI conditions, such varieties improved their must antioxidant properties, which probably was related to the higher extractable anthocyanins of TV (Figure 4) and higher total phenolic compounds of GRA (Figure 5), respectively, in ETDI compared with the control conditions, ATFI [55,56]. 

It has been suggested that the application of DI can help to synchronize the technological and phenolic maturity of grapes [48]. In our study, when comparing ATFI with ATDI, this affirmation seems to be true for TV (higher anthocyanin content) (Figure 4) and GRA (higher extractable anthocyanins and antioxidant capacity) (Figure 5 and Figure 7). Although some studies have suggested that the benefits of DI on berry quality could be lost under warming temperatures [23,24], this did not occur in the present study. Thus, in TV, the enhancement in the content of anthocyanins detected under ATDI treatment was also achieved under ETDI, whereas in GRA, ETDI conditions even improved berry phenolic content and the potential of anthocyanin extraction. Taken together, the findings of this study support those presented in Antolín et al. [35] and underline the interest in assessing the performance of ancient grapevine varieties under multiple environmental stress factors and their physiological potential to cope with environmental constraints.

## 4. Materials and Methods

### 4.1. Biological Material and Growth Conditions

The grapevine (*Vitis vinifera* L.) varieties included in this study were selected from more than 65 genotypes recovered, from 2002 to 2020, in old vineyards (older than 65 years) that were identified using molecular markers [36,57], and multiplied and conserved in the germplasm bank of the Estación de Viticultura y Enología de Navarra (EVENA) (Navarra, Spain). Genetic profiles of the four varieties are presented in Supplementary Table S1. The varieties were selected on the basis of agronomic characterization, realized by the EVENA [57]. The four genotypes were grown in an experimental vineyard located in Olite (Navarra, Spain) (latitude: 42°29′15″ N; longitude: 1°39′45″ W; altitude: 388 m.a.s.l.). One hundred dormant cuttings of each genotype were collected after the winter pruning of 2020. 

The 400–500 mm long cuttings were prepared for fruit-bearing following the methodology developed by Mullins [58], which consists of inducing rooting by immersing the cuttings in a solution of indole-3-butyric acid (400 mg L^−1^) and placing them in a warm bed (27 °C) in a cold room (4 °C) for 30 days. After rooting was successful, the cuttings were planted in 0.8 L plastic pots containing perlite and peat (1:1 *v*:*v*) and then, they were transferred to a greenhouse where the environmental conditions were 25/15 °C and 50/90% relative humidity (day/night) regime and natural daylight (photosynthetic photon flux density, PPFD, was on average 850 μmol m^−2^ s^−1^ at midday) supplemented with high-pressure sodium lamps (OSRAM, Augsburg, Germany) to increase the photoperiod to 15 h with a minimum PPFD of 350 μmol m^−2^ s^−1^ at the inflorescence level. After 7–8 days bud-break took place and from this moment to flowering plant growth was controlled to leave one inflorescence and 4 leaves per plant. While flowering in Tempranillo (TEM), Tinto Velasco (TV), Graciano (GRA), and Grand Noir (GNO) started, respectively, on 4, 7 and 9 May 2021, the fruit set occurred on 11, 14 and 16 May 2021. 

### 4.2. Experimental Design

After the fruit set (Eichhorn and Lorenz (E-L) growth stage 27) [59], which took place about one month after the bud-break, plants were transplanted to 13 L plastic pots containing perlite and peat (1:1 *v*:*v*). Plants grew freely until reaching 16 leaves per plant, and then, the plants were regularly pruned to maintain an appropriate leaf area to fruit mass ratio for berry ripening [60]. Subsequently, plants were moved to four temperature gradient greenhouses (TGG) located at the University of Navarra (Pamplona, Spain) (latitude: 42°49′00″ N; longitude: 1°39′00″ W; altitude: 450 m.a.s.l.) for the application of the different temperature regimes. TGGs have a modular design with three temperature modules (3.04 m long each) which maintain a temperature gradient, from module 1 (ambient temperature) to module 3 (ambient temperature + 4 °C), by circulating air to maintain a temperature difference of 4 °C between modules [61]. No plants were placed into module 2 because their temperature is not stable. Inside the TGG, the pots were placed in holes made in the soil to simulate natural temperature fluctuations, thus keeping the temperature differences between shoots and roots found under field conditions [62]. Plants of all varieties were randomly distributed into the TGG, and two temperature conditions were set up from the fruit set: (1) ambient temperature (AT) and elevated temperature (ambient + 4 °C, ET). 

When berries began to color and enlarge (E-L 35 stage, veraison), plants within each temperature regime were subdivided into two groups that were subjected to different irrigation schedules: (1) plants under full irrigation (FI), and (2) plants that received 50% of the water given to FI plants from veraison to maturity (E–L 38 stage) (deficit irrigation, DI). Soil moisture sensors (EC-5 Soil Moisture Sensors, Decagon Devices Inc., Pullman, WA, USA) were placed in each pot. FI plants were maintained at ca. 80% of pot capacity (sensor value between 30 and 35%, (m^3^ H_2_O m^−3^ soil) × 100). In the DI treatment, plants were watered when the sensor value reached 50% of the value of FI (15–20%, (m^3^ H_2_O m^−3^ soil) × 100) from veraison to maturity (E-L 38 stage). Previously, pot capacity was determined as water retained after free-draining water had been passed through the holes in the bottom of the pot. Plants were watered twice a day with irrigation doses ranging from 2.5 L to 3.5 L per pot according to the plant’s needs according to the daily measurements of the EC 5 water sensor. The irrigation (both at the pre-treatment greenhouse and the TGGs) was carried out using a nutritive solution in accordance with viticultural requirements [63] alternated with water. Quartz stones were added to the surface of the pots to reduce losses of water from the substrate by evaporation. 

Altogether, a two-factorial design was established where two temperature regimes were combined with two water regimes, setting up four treatments: (1) ambient temperature (T) and full irrigation (ATFI); (2) ambient temperature and deficit irrigation (ATDI); (3) elevated temperature (T + 4 °C) and full irrigation (ETFI), and (4) elevated temperature and deficit irrigation (ETDI). There were 5 biological replicates (plants) for each combination of temperature, irrigation, and variety. Plants were kept in the TGGs until a commercial maturity of berries was attained (E-L 38 stage). 

### 4.3. Weather Conditions 

The minimum, mean, and maximum daily temperatures were registered throughout the experiment. Weather data were obtained from the Pamplona Airport station (Navarra, Spain) and the reference period from 2001 to 2021 [64]. Data show that the growing season of 2021 was warmer than that of the same period within the last 20 years (Table 5), with the maximum daily air temperature being between 2.5 and 4 °C higher than the average recorded for the 2001–2021 period. 

Particularly, September was warmer with the maximum and minimum daily air temperatures being 2.5 and 2 °C higher than the average registered for the same month during the period 2001–2021. Moreover, treatments of ambient (AT) and elevated temperature (ET) experienced 11 and 24 days, respectively, at a maximum temperature above 35 °C (Table 2). It is considered a heatwave, a period of five consecutive days with maximum daily air temperatures above 35 °C [65]. According to this definition, three and one heatwaves were recorded during the ET and AT treatments, respectively.

### 4.4. Plant Measurements

Predawn leaf water potential (Ψ_pd_) was measured with a SKYE SKPM 1400 pressure chamber (Skye Instruments Ltd., Llandrindod, Wales, UK) on fully expanded leaves just before irrigation. Determinations were made at three stages of berry ripening: (1) seven days after veraison (E–L 36 stage); (2) fourteen days after veraison (E–L 37 stage) and (3) fruit maturity (E-L 38 stage). Afterwards, every plant was harvested when the ratio of sugars to acidity ranged between 4 and 6. The main phenological phases of berry ripening were registered as the number of days from fruit set (E–L 27 stage) to veraison (E–L 35 stage), and from veraison (E–L 35 stage) to maturity (E–L38 stage). 

At harvest, all leaves were removed, and leaf area was measured with a portable area meter (model LI-3000, Li-Cor, Lincoln, NE, USA). Afterwards, the length and weight of bunches were recorded, and the bunches were destemmed to record the number of berries and total berry weight. Bunch compactness was calculated as the quotient between the weight and the squared length of the bunch. From each plant, 10 berries were separated into skin and flesh to calculate the relative skin mass as the quotient between skin fresh matter (FM) and whole berry FM. The rest of the berries were stored at −20 °C for subsequent analyses. 

### 4.5. Technological Maturity

Parameters included in the technological maturity were berry total soluble solid (TSS) concentration (expressed as °Brix), pH, and total acidity (expressed in g L^−1^ tartaric acid). For each plant, a sample of 20 berries was pressed and extracts were centrifuged at 4100× *g* at 4 °C for 10 min. The supernatant was used for the following determinations: TSS measured with a temperature-compensating refractometer (Zuzi model 315; Auxilab, Beriáin, Spain); must pH measured with a pH meter (Crison Instruments, Barcelona, Spain) standardized to pH 7.0 and 4.0; titratable acidity measured by titration with NaOH [66]. 

### 4.6. Phenolic Maturity

Grape phenolic maturity parameters included total and extractable anthocyanins, cellular extractability (EA) of anthocyanins, total polyphenol index (TPI) and phenolic seed maturity (SM). A 20-berry sample per plant was utilized for the analysis of anthocyanins, total phenols, and chromatic properties. For berry anthocyanin extraction, two samples of the non-filtered, triturated berry homogenate were macerated for 4h at pH 1 (hydrogen chloride) and pH 3.2 (tartaric acid), respectively [67]. Then, the macerated samples were centrifuged at 4100× *g* at 4 °C for 10 min. Total and extractable anthocyanins were determined in both supernatants (macerated at pH 1 and pH 3.2) by reading absorbance at 520 nm after reaction with bisulfite [68]. The pH 1.0 extraction produces the degradation of the skin cells, thus leading to the liberation, diffusion, and solubilization of the most percentage of the phenolic compounds. The pH 3.2 extraction procedure is similar to the one used during a classic vinification [67]. The cellular extractability of anthocyanins (EA) represents the percentage of extractable anthocyanins at a pH of 3.2 upon the maximum possible, extracted at a pH of 1.0 [53]. 

The total polyphenol index (TPI) was estimated from the absorbance reading at 280 nm in the supernatant obtained after maceration at pH 3.2 [69]. During berry maturation, the concentration of tannins in the seeds decreases and their contribution to the phenolic concentration is lower [67]. Thus, the phenolic maturity of seeds (SM) represents the contribution percentage of the seed tannins to the wine phenolic richness and was calculated according to the Glories procedure [53]. Finally, color density was calculated as the sum of the absorbance readings at 420, 520, and 620 nm of the samples extracted at pH 3.2, and the tonality index was obtained from the quotient of absorbance readings at 420 and 520 nm [70]. 

### 4.7. Total Antioxidant Capacity

Total antioxidant capacity was analyzed on the must samples used for technological maturity determinations by using the free-radical scavenging activity (α, α-diphenyl-β-picrylhydrazyl, DPPH) assay [71]. The reaction was started by adding 25 µL of the sample to the medium containing 80 µM (methanol solution) (975 µL) of the free radical (DPPH•). Samples were incubated for 15 min at 25 °C, after which the absorbance readings at 515 nm were recorded. The calibration curve was made using gallic acid as a standard and results were expressed as gallic acid equivalents (mg L^−1^). 

### 4.8. Statistical Analyses

Statistical analyses were carried out using the Statistical Package for the Social Sciences (SPSS) software (SPSS Inc., Chicago, IL, USA) version 21.0 for Windows. After establishing that the data met the assumptions of normality (Shapiro–Wilk’s test) and homoscedasticity (Levene’s test) with a threshold of 0.05, two and three-way analyses of variance (ANOVA) were performed to assess the main effect of the factor variety, temperature, and irrigation level, and the interaction between them. When ANOVA was statistically significant (*p* < 0.05), the differences among groups were tested with Duncan’s posthoc test. Results were considered statistically significant if *p* < 0.05. 

## 5. Conclusions

This study provides evidence for the diversity of ancient grapevine varieties resulting in different abilities to cope with increasing temperature and deficit irrigation. Beyond the common responses to high temperature and deficit irrigation that arise from the present study, significant differences among the varieties studied have been found in plant water relations, technological and phenolic maturity, and antioxidant properties of must. Under our experimental conditions, GNO was the most sensitive variety to the environmental conditions since elevated temperature and water deficit, acting separately or in combination increased must pH, and reduced titratable acidity and anthocyanin content. By contrast, varieties such as TV and GRA were superior under deficit irrigation and warm temperatures in terms of fruit quality (mainly, anthocyanins and total phenolic content), oenological potential, and antioxidant properties indicating that such two minor varieties stand out as alternatives to widespread varieties to be exploited. Our study underlines the importance of evaluating the behavior of the ancient grapevine varieties that could offer good options for the adaptation of viticulture to future climate conditions. However, studies carried out under controlled conditions in potted young plants have limitations to interpretation. Further studies under natural conditions are required before extrapolating the present results to field-grown ancient varieties tested. Moreover, the selection of resilient grapevine varieties to environmental constraints should be combined with adequate rootstocks whose root system architecture and root transcriptomic regulations make them tolerant to abiotic stresses. 

## Figures and Tables

**Table 1:** Local ancient grapevine varieties studied. Cuttings were collected during the winter of 2020 from plants grown in the vineyard located in the Estación de Viticultura y Enología de Navarra (EVENA) (Navarra, Spain).

	Clone	Maturation Cycle	Colour	Code
Tempranillo	T24	Short	Red	TEM
Tinto Velasco	T73	Medium	Red	TV
Graciano	T72	Medium	Red	GRA
Grand Noir	T48	Medium	Teinturier	GNO

**Table 2:** General characteristics of fruit-bearing cuttings of different ancient wine grape varieties grown either at ambient (AT) or elevated temperature (ET) and subjected to different irrigation regimes (FI, full irrigation; DI, deficit irrigation). The variety labels can be found in Table 1.

	TEM	TEM	TV	GRA	GNO
Phenology	Fruit set–veraison (d)	53 c	58 b	62 a	55 c
	Veraison–maturity (d)	51 a	52 a	45 b	53 a
Plant growth	Leaf area (m^2^ plant^−1^)	0.32 b	0.67 a	0.40 b	0.37 b
	Leaf size (cm^2^ leaf^−1^)	202.8 b	363.4 a	269.5 b	215.3 b
Bunch traits	Bunch mass (g FM bunch^−1^)	182.4 a	108.5 b	74.9 c	95.0 bc
	Bunch compactness (g cm^−2^)	0.68 b	1.01 a	0.64 b	0.86 a
	Berries (number bunch^−1^)	158 a	155 a	90 b	92 b
Berry traits	Berry mass (g FM berry^−1^)	1.12 a	0.57 c	0.69 c	0.94 b
	Seeds (number berry^−1^)	2 a	1 b	1 b	1 b
	Relative skin mass (% berry FM)	26.4 a	27.8 a	26.9 a	25.5 a ^1^

^1^ Values represent means (*n* = 20). For each variety, the data of all treatments have been joined to evaluate the effect of the variety as the main factor. Within rows, means followed by the same letter do not differ significantly (*p* ≥ 0.05) according to Duncan’s test. FM: fresh matter.

**Table 3:** Phenology, plant growth, and fruit characteristics from fruit-bearing cuttings of different ancient wine grape varieties grown either at ambient (AT) or elevated temperature (ET) and subjected to different irrigation regimes (FI, full irrigation; DI, deficit irrigation). The variety labels can be found in Table 1.

		**TEM**						
		**Treatments**				**ANOVA**		
		**ATFI**	**ATDI**	**ETFI**	**ETDI**	**T**	**I**	**T × I**
Phenology	Fruit set–veraison (d)	55 a	52 a	52 a	52 a	ns	ns	ns
	Veraison–maturity (d)	44 a	51 a	56 a	55 a	ns	ns	ns
Plant growth	Leaf area (m^2^ plant^−1^)	0.40 a	0.28 a	0.35 a	0.27 a	ns	ns	ns
	Leaf size (cm^2^ leaf^−1^)	270.4 a	186.0 a	191.5 a	163.4 a	ns	ns	ns
Bunch traits	Bunch mass (g FM bunch^−1^)	204.6 a	169.5 a	190.4 a	165.1 a	ns	ns	ns
	Bunch compactness (g cm^−2^)	0.73 a	0.77 a	0.63 a	0.61 a	ns	ns	ns
	Berries (no bunch^−1^)	175 a	127 a	168 a	162 a	ns	ns	ns
Berry traits	Berry mass (g FM berry^−1^)	1.11 a	1.26 a	1.11 a	1.00 a	ns	ns	ns
	Seeds (no berry^−1^)	2 a	2 a	2 a	2 a	ns	ns	ns
	Relative skin mass (% berry FM)	24.7 b	21.0 b	25.9 ab	33.9 a	*	ns	ns
		**TV**						
		**Treatments**				**ANOVA**		
		**ATFI**	**ATDI**	**ETFI**	**ETDI**	**T**	**I**	**T × I**
Phenology	Fruit set–veraison (d)	58 a	58 a	57 a	57 a	ns	ns	ns
	Veraison–maturity (d)	57 a	51 ab	51 ab	47 b	*	*	ns
Plant growth	Leaf area (m^2^ plant^−1^)	0.69 a	0.61 ab	0.56 ab	0.44 b	*	ns	ns
	Leaf size (cm^2^ leaf^−1^)	416.5 a	342.8 a	399.7 a	312.2 a	ns	ns	ns
Bunch traits	Bunch mass (g FM bunch^−1^)	135.2 a	144.9 a	60.1 b	93.8 ab	**	ns	ns
	Bunch compactness (g cm^−2^)	0.68 b	1.20 a	0.69 b	1.49 a	ns	***	ns
	Berries (no bunch^−1^)	140 a	193 a	144 a	142 a	ns	ns	ns
Berry traits	Berry mass (g FM berry^−1^)	0.71 a	0.57 a	0.49 a	0.51 a	ns	ns	ns
	Seeds (no berry^−1^)	1 a	1 a	1 a	1 a	ns	ns	ns
	Relative skin mass (% berry FM)	31.2 a	23.3 a	29.5 a	27.2 a	ns	ns	ns
		**GRA**						
		**Treatments**				**ANOVA**		
		**ATFI**	**ATDI**	**ETFI**	**ETDI**	**T**	**I**	**T × I**
Phenology	Fruit set–veraison (d)	63 a	60 a	63 a	61 a	ns	ns	ns
	Veraison–maturity (d)	45 a	45 a	43 a	46 a	ns	ns	ns
Plant growth	Leaf area (m^2^ plant^−1^)	0.44 ab	0.37 ab	0.52 a	0.29 b	ns	*	ns
	Leaf size (cm^2^ leaf^−1^)	315.4 a	224.5 a	317.7 a	220.2 a	ns	ns	ns
Bunch traits	Bunch mass (g FM bunch^−1^)	90.6 a	84.9 a	81.1 a	43.1 b	*	ns	ns
	Bunch compactness (g cm^−2^)	0.62 a	0.68 a	0.66 a	0.58 a	ns	ns	ns
	Berries (no bunch^−1^)	102 a	98 a	95 ab	65 b	*	ns	ns
Berry traits	Berry mass (g FM berry^−1^)	0.82 a	0.69 a	0.76 a	0.49 b	*	**	ns
	Seeds (no berry^−1^)	1 a	1 a	1 a	1 a	ns	ns	ns
	Relative skin mass (% berry FM)	26.9 a	25.3 a	26.9 a	28.3 a	ns	ns	ns
		**GNO**						
		**Treatments**				**ANOVA**		
		**ATFI**	**ATDI**	**ETFI**	**ETDI**	**T**	**I**	**T × I**
Phenology	Fruit set–veraison (d)	55 a	56 a	54 a	54 a	ns	ns	ns
	Veraison–maturity (d)	52 a	51 a	56 a	53 a	ns	ns	ns
Plant growth	Leaf area (m^2^ plant^−1^)	0.47 ab	0.24 b	0.53 a	0.26 b	ns	**	ns
	Leaf size (cm^2^ leaf^−1^)	274.3 a	146.4 b	274.1 a	166.3 b	ns	**	ns
Bunch traits	Bunch mass (g FM bunch^−1^)	97.05 a	96.5 a	86.1 a	100.2 a	ns	ns	ns
	Bunch compactness (g cm^−2^)	0.77 a	0.91 a	0.88 a	0.87 a	ns	ns	ns
	Berries (no bunch^−1^)	103 a	103 a	66 b	95 ab	*	ns	ns
Berry traits	Berry mass (g FM berry^−1^)	0.89 a	0.84 a	1.09 a	0.96 a	ns	ns	ns
	Seeds (no berry^−1^)	1 a	1 a	1 a	1 a	ns	ns	ns
	Relative skin mass (% berry FM)	29.9 a	24.9 ab	24.0 ab	23.1 b	ns	ns	ns ^1^

^1^ Values represent means (*n* = 5). Within rows, means followed by the same letter do not differ significantly (*p* ≥ 0.05) according to Duncan’s test as affected by the main factors temperature (T), irrigation (I), and their interaction (T × I). Significance of the analysis of variance (ANOVA): * *p* < 0.05; ** *p* ≤ 0.01; *** *p* ≤ 0.001; ns, not significant (*p* ≥ 0.05). FM: fresh matter.

**Table 4:** Main effects and their interactions on berry quality and antioxidant capacity from fruit-bearing cuttings of different ancient wine grape varieties grown either at ambient (AT) or elevated temperature (ET) and subjected to different irrigation regimes (FI, full irrigation; DI, deficit irrigation). The variety labels can be found in Table 1.

Main Effects	TSS (°Brix)	Must pH	Titratable Acidity (g L^−1^)	Color Density (AU)	Tonality Index	TPI (AU)	Total Anthocyanins (mg L^−1^)	Antioxidant Capacity (mg L^−1^)
Variety (V)								
TEM	20.4 a	4.09 a	5.40 a	3.56 c	0.56 b	33.1 c	375.9 c	22.6 ab
TV	19.9 b	3.82 c	5.93 a	3.87 c	0.68 a	32.6 c	285.0 d	21.5 ab
GRA	21.2 ab	3.70 c	5.68 a	7.78 a	0.65 a	44.9 b	527.1 b	20.9 b
GNO	21.0 ab	3.96 b	4.24 b	6.86 b	0.57 b	53.0 a	629.4 a	23.9 a
Temperature (T)								
AT	21.6 a	3.78 b	5.69 a	5.61 a	0.59 b	39.8 a	488.6 a	21.8 a
ET	20.7 a	4.00 a	4.93 b	5.42 a	0.64 a	42.0 a	420.2 b	22.6 a
Irrigation (I)								
FI	20.4 b	3.83 b	5.32 a	5.51 a	0.62 a	40.5 a	457.2 a	21.6 a
DI	21.9 a	3.96 a	5.31 a	5.53 a	0.61 a	41.3 a	451.5 a	22.8 a
ANOVA								
Variety (V)	*	***	***	***	***	***	***	**
Temperature (T)	ns	***	***	ns	**	ns	**	ns
Irrigation (I)	*	***	ns	ns	ns	ns	ns	ns
V × T	ns	ns	ns	**	ns	**	***	*
V × I	ns	ns	**	ns	*	*	**	ns
T × I	ns	ns	ns	ns	ns	ns	ns	ns
V × T × I	ns	ns	***	ns	ns	ns	ns	ns ^1^

^1^ Values represent means. Within columns, means followed by the same letter do not differ significantly (*P* ≥ 0.05) according to Duncan’s test as affected by the main factors variety (V) (*n* = 20), temperature (T) (*n* = 40) irrigation (I) (*n* = 40), and their interactions. Significance of the analysis of variance (ANOVA): * *p* < 0.05; ** *p* ≤ 0.01; *** *p* ≤ 0.001; ns, not significant (*p* ≥ 0.05). TSS, total soluble solids; TPI, total polyphenol index; AU, absorbance units.

**Table 5:** Weather conditions during the growing season of 2021 and the average for the same period in the last 20 years (2001–2021). The number of days with temperatures above 35 °C was registered in the ambient (AT) and elevated temperature (ET) modules of the TGG, respectively.

Month	Year	Mean Daily Air Temperature (°C)	Minimum Daily Air Temperature (°C)	Maximum Daily Air Temperature (°C)	Number of Heat Days (>35 °C) (AT)	Number of Heat Days (>35 °C) (ET)
June	2021	18.0	8.0	33.0	0	0
	2001–2021	19.6	7.5	35.4		
July	2021	20.0	9.0	41.0	5	9
	2001–2021	21.5	10.0	37.0		
August	2021	20.0	9.0	41.0	5	10
	2001–2021	21.7	9.9	37.3		
September	2021	19.0	8.0	35.0	1	5
	2001–2021	18.6	6.0	32.6 ^1^		

^1^ Weather data recorded from the Pamplona Airport station (Navarra, Spain) were provided AEMET [64].

## Data Availability

Data files of the present study have been deposited in the Department of Environmental Biology (University of Navarra).

## Supplementary Materials

The following supporting information can be downloaded at: https://www.mdpi.com/article/10.3390/plants11212929/s1, Table S1: Code, passport number, variety name, origin, genotype, and molecular profiles of ancient grapevine varieties.
