# Supplementary material for: Prospecting the Resilience of Several Spanish Ancient Varieties of Red Grape under Climate Change Scenarios
Source: Plants (Basel). 2022 Oct 31;11(21):2929. doi: 10.3390/plants11212929 (PMC9653837; doi:10.3390/plants11212929)
Supplement: Supplementary file 1 [file plants-11-02929-s001.zip › plants-1999568-supplementary.pdf]

Table S1. Code, passport number, variety name, origin, genotype, and molecular profiles (allele sizes in base pairs) of ancient grapevine varieties identified with 8 microsatellite markers.

Code	Passport	Variety	Origin	Genotype	VMC4F31		VVIN16		VVIV37		VVIV67		VVMD27		VVIP31		VVS2		ZAG79	
T24	CS0024	Tempranillo	Bargota (Navarra)	GEN 0055	179	183	151	153	171	171	366	368	183	183	178	180	141	144	247	251
T73	CS0073	Tinto Velasco	Los Arcos (Navarra)	GEN 0062	187	206	151	151	158	158	358	375	179	185	184	190	131	131	237	251
T72	CS0072	Graciano	Los Arcos (Navarra)	GEN 0045	179	206	151	159	165	177	358	364	179	183	180	192	137	151	251	259
T48	CS0048	Grand Noir	Los Arcos (Navarra)	GEN 0157	173	179	151	153	163	177	358	364	181	183	176	180	137	151	243	259
